# An Appetite for Modernizing the Regulatory Framework for Protein Content Claims in Canada

**DOI:** 10.3390/nu9090921

**Published:** 2017-08-23

**Authors:** Christopher P. F. Marinangeli, Samara Foisy, Anna K. Shoveller, Cara Porter, Kathy Musa-Veloso, John L. Sievenpiper, David J. A. Jenkins

**Affiliations:** 1 Pulse Canada, 1212-220 Portage Avenue, Winnipeg, MB R3C 0A5, Canada; 2 Loblaw Companies Limited, 1 President’s Choice Circle, Brampton, ON L6Y 5S5, Canada; samara.foisy@loblaw.ca (S.F.); Cara.Porter@loblaw.ca (C.P.); 3 Department of Animal Biosciences, University of Guelph, 50 Stone Road East, Guelph, ON N1G 2W1, Canada; ashovell@uoguelph.ca; 4 Intertek Scientific & Regulatory Consultancy, 2233 Argentia Road-Suite 201, Mississauga, ON L5N 2X7, Canada; kathy.musa-veloso@intertek.com; 5 Department of Nutritional Sciences, University of Toronto, Toronto, ON M5B 1W8, Canada; john.sievenpiper@utoronto.ca (J.L.S.); david.jenkins@utoronto.ca (D.J.A.J.); 6 Clinical Nutrition & Risk Factor Modification Center, St. Michael’s Hospital, Toronto, ON M5B 1W8, Canada; 7 Department of Medicine, Division of Endocrinology and Metabolism, St. Michael’s Hospital, Toronto, ON M5B 1W8, Canada; 8 Li Ka Shing Knowledge Institute, St. Michael’s Hospital, Toronto, ON M5B 1W8, Canada

**Keywords:** protein, protein quality, protein efficiency ratio, protein digestibility corrected amino acid score (PDCAAS), regulation

## Abstract

The need for protein-rich plant-based foods continues as dietary guidelines emphasize their contribution to healthy dietary patterns that prevent chronic disease and promote environmental sustainability. However, the Canadian *Food and Drug Regulations* provide a regulatory framework that can prevent Canadian consumers from identifying protein-rich plant-based foods. In Canada, protein nutrient content claims are based on the protein efficiency ratio (PER) and protein rating method, which is based on a rat growth bioassay. PERs are not additive, and the protein rating of a food is underpinned by its Reasonable Daily Intake. The restrictive nature of Canada’s requirements for supporting protein claims therefore presents challenges for Canadian consumers to adapt to a rapidly changing food environment. This commentary will present two options for modernizing the regulatory framework for protein content claims in Canada. The first and preferred option advocates that protein quality not be considered in the determination of the eligibility of a food for protein content claims. The second and less preferred option, an interim solution, is a framework for adopting the protein digestibility corrected amino acid score as the official method for supporting protein content and quality claims and harmonizes Canada’s regulatory framework with that of the USA.

## 1. Introduction

Protein quality refers to the ability of a dietary protein to meet the metabolic demands of a human and largely depends on levels of indispensable amino acids and their digestibility within a food matrix [1]. In Canada, to claim that a food is a source of protein, the food must meet thresholds for protein quality using the protein efficiency ratio (PER) and protein rating methodology. The PER is generated from a bioassay where the growth rate of rats consuming the test protein is compared to the growth rate of rats consuming casein as a dietary source of protein. The protein rating and the eligibility for a “source” of protein claim is determined by multiplying the quantity of protein present in a Reasonable Daily Intake (RDI) of the food by the PER. Unfortunately, the PER and protein rating methodology have significant limitations: 1. The growing rat is not considered to be a good model for determining the amino acid requirements of humans, particularly adults; 2. The inability to combine PER values; and 3. reliance on RDIs for determining a protein rating. Given these limitations, the current regulatory framework for protein claims in Canada is a barrier to aligning with global initiatives aimed at increasing the prominence of plant sources of protein in healthy dietary patterns that also help to address issues around environmental sustainability and food production.

As plant-based foods with significant levels of protein gain more prevalence in dietary guidelines for their contribution to healthy dietary patterns that prevent chronic disease and promote environmental sustainability, regulatory frameworks should enable consumers to easily identify plant-based foods that are sources of protein. Dietary guidelines in the Netherlands suggest that increased consumption of plant-based foods reduces the risk of chronic disease and promotes environmental sustainability [2]. These sentiments are echoed by the 2012 Nordic Nutrition Recommendations [3], and USA Scientific Report of the 2015 Dietary Guidelines Advisory Committee [4]. Similarly, the French Agency for Food, Environmental and Occupational Health & Safety recently recommended that the prominence of plant-based sources of protein, particularly pulses, be increased in the next iteration of France’s food-based dietary guidelines of the National Health and Nutrition Programme [5]. With a growing world population and increasing environmental and animal welfare concerns, the sustainability of current food production systems requires that food systems increase the appeal of alternative protein sources [6,7,8]. Canada’s ongoing use of the PER/protein rating method highlights a missed opportunity to educate Canadians on alternative plant-based sources of protein.

Canada is not the only region to use protein quality to underpin protein nutrient content claims for the labelling and advertising of foods. The USA for example, uses the protein digestibility corrected amino acid score (PDCAAS) to evaluate the protein quality of foods targeted to children (≥1 year of age) through to adults. To account for protein quality, the PDCAAS method adjusts the level of protein in a food by using the indispensable amino acid content of the food (mg/g protein), indispensable amino acid requirements of a reference population (mg/g protein), and the weighted average true fecal nitrogen digestibility of the sources of protein in the test food [9]. The true fecal nitrogen digestibility of foods is determined in vivo using humans or the rat model [9]. However, other regions, such as Europe [10], Australia and New Zealand [11], China [12], and South Korea [13] use absolute thresholds to support protein content claims on foods that are based on the level of protein (g) per serving, the proportion of energy from protein per serving, and/or the level of protein per unit of energy.

The purpose of this commentary is to provide suggestions to expedite the modernization of the Canadian *Food and Drug Regulations* that govern protein content claims in Canada and the ability to communicate the presence of protein in a food meaningfully to Canadians. Following brief summaries of the PER/protein rating and PDCAAS methodologies, the challenges that the ongoing use of the PER and corresponding protein rating system present for the Canadian food system will be discussed and two options for modernizing the regulatory framework for protein content claims are presented. The first and most preferred option (Option 1) is to allow protein content claims in food labeling and advertising in Canada to be based on the absolute protein content and not based on protein quality. This option is the most agile and presents minimal barriers for the Canadian food system to adapt and align with evolving dietary guidelines and recommendations. Option 1 also harmonizes with current regulatory frameworks in other key developed jurisdictions globally, including Europe, Australia and New Zealand, China, and South Korea. The second and less-preferred option (Option 2) is for Canada to adopt a modified version of the PDCAAS-based framework as the official method for supporting protein claims. While this approach is more restrictive than the first option, adoption of PDCAAS would harmonize Canada’s regulatory framework for protein content claims with that of the USA. Modifications for the proposed Canadian PDCAAS framework includes the use of in vitro methods to determine true fecal nitrogen digestibility and adoption of a 3-tier claim hierarchy. The suggested options discussed in this commentary will reduce some of the barriers for identifying plant-based protein sources.

## 2. Summary of Current Regulatory Frameworks for Protein Content Claims in Canada and the USA

### 2.1. Summary of the PER and Protein Rating Methodologies for Supporting Protein Content Claims in Canada

**Figure 1:**
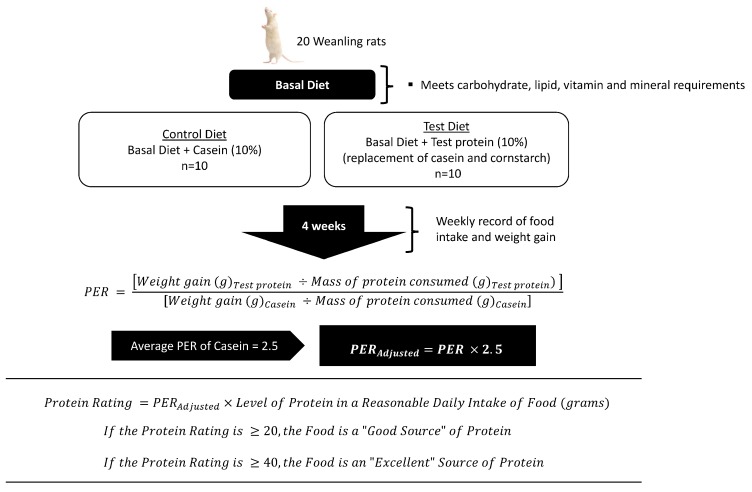
Canadian regulatory framework for protein content claims where the adjusted PER is used to generate a protein rating for a given food [16]. The PER of 2.5 represents the standardized protein rating for casein. It is used to account for intra-laboratory variation and normalize PER values that are generated by the in vivo rat bioassay [14]. PER, protein efficiency ratio. Figure 1 was adapted from Marinangeli and House [15].

Originally developed in the early 20th century, the PER was the first method adopted for the routine assessment of the protein quality of foods. Given that the PER is woven into Canada’s *Food and Drug Regulations*, Health Canada has made Method FO-1 available, which outlines the study protocol for measuring the adjusted PER for a food and its corresponding protein rating [14]. This method is summarized in Figure 1 [15]. Briefly, a test diet and a casein control diet, both containing 10% protein (N × 6.25), are fed to weanling rats for a period of 4 weeks. The PER values are calculated by dividing the weight gain of the rats by the amount of protein consumed over this period. To correct for intra-laboratory variation, the generated PER values are corrected to an assumed PER value of 2.5 for a casein control [14]. For the purposes of this discussion, the term “PER” refers to the adjusted PER value. Once the PER for a food is established, its protein rating is the product of the PER multiplied by the level of protein in an RDI of the food in question. A Food with a protein rating of ≥20 is considered a “good source,” while a food with a protein rating of ≥40 is considered an “excellent source” of protein [16]. RDIs for various foods are listed in Schedule K of the Canadian *Food and Drug Regulations* [17]. When an RDI for a food does not exist, the reference amount can be used [18].

### 2.2. Summary of the PDCAAS Methodology for Supporting Protein Content Claims in the USA

**Figure 2:**
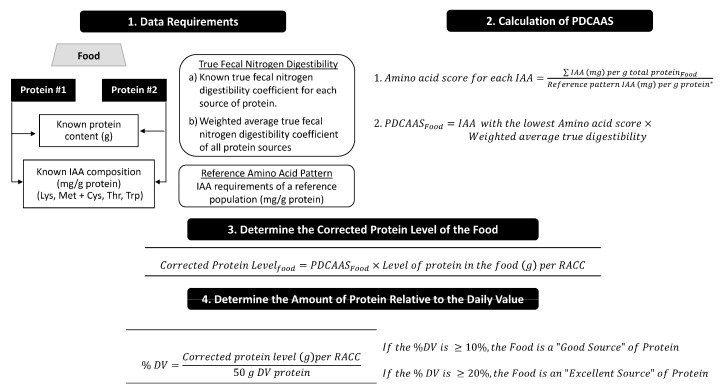
USA regulatory framework for protein content claims where PDCAAS and a 50 g DV for protein are used to determine if a food qualifies for a protein content claim [19]. This method applies to foods marketed to children ≥4 years through to adults. Only IAAs that are low particularly in plant-based foods are listed. DV, Daily Value; IAA, indispensable amino acid; Lys, lysine; Met + Cys, methionine + cysteine; PDCAAS, protein digestibility corrected amino acid score; RACC, reference amount customarily consumed; Thr, threonine; Trp, tryptophan. Figure 2 was adapted from Marinangeli and House [15].

Rather than PER, PDCAAS is used in the USA as a measure of protein quality and formulates the basis for protein content claims for foods marketed to those ≥1 year of age [19]. Compared to PER, PDCAAS is recognized as a more accurate characterization of the protein quality of foods for human consumption [20,21]. As summarized in Figure 2 [15], PDCAAS is determined using the total fecal nitrogen digestibility value and indispensable amino acid concentrations (mg/g protein) of protein sources within a given food [9,22]. If human data are unavailable, total fecal digestibility of a protein source is determined using the rat balance method [9]. When protein digestibility is considered, the ratio of indispensable amino acid concentrations in the food to the indispensable amino acids requirements of the reference population is calculated. The Food and Drug Administration (FDA) has outlined that the scoring pattern corresponding to preschool children (2–5 years) as summarized in the 1991 Report of the Joint Food and Agriculture Organization of the United nations/World Health Organization (FAO/WHO) Expert Consultation on Protein Quality [9] should be utilized [19]. The lowest ratio for the indispensable amino acids is multiplied by true fecal nitrogen digestibility and is considered the PDCAAS for the food. PDCAAS values that are >1 are truncated at 1. True fecal nitrogen digestibility values can be attained from a variety of sources including documentation from the FAO [9], the peer-reviewed literature, or is independently assessed by food industry stakeholders.

For children (≥4 years of age) through to adults, protein content claims in the USA are based on the corrected protein content within a food relative to the Daily Value (DV) for protein (50 g) (Figure 2) [19]. If the corrected protein levels are ≥10% (5 g) and ≥20% (10 g) of the DV per reference amount customarily consumed (RACC), the food can be considered a “good” and “excellent” source of protein, respectively [19]. The protein DV for children 1 through 3 years of age is 13 g. It is also important to note that foods intended for infants (≤12 months of age) are required to use the PER method for determining protein quality and eligibility for a protein claim [19]. While the exact reasons are unclear, at the time when food labeling regulations were adopted in the USA, ongoing use of the PER for infant foods could stem from the lack of certainty with respect to the amino acid requirements of the growing infant and the knowledge that, during infancy, a significant proportion of protein is used for growth [9]. In contrast, from childhood through to adulthood, the majority of protein is used for maintenance [9].

## 3. Current Challenges Associated with using the PER and Protein Rating System to Support Protein Content Claims in Canada

### 3.1. Challenge 1: Methodological Assumptions of the PER

Although simple, it has been recognized that the PER method is not a valid bioassay to decipher nutritional protein quality recommendations for humans [9]. These failures were discussed in the 1991 FAO report, “Protein Quality Evaluation: Report of Joint FAO/WHO Expert Consultation,” and is predominantly due to the fact that the amino acid requirements of rats differ from those of humans. In particular, the sulphur amino acid requirements of the growing rat are much higher than the human [9]. Thus, when a food has limited levels of sulphur amino acids, as is the case for legumes (pulses, peanuts and soybeans), the PER assay will underestimate protein quality [9]. Use of the growing rat model does not fully credit those amino acids that would be used for maintenance [9]. Additionally, animal care protocols that require prolonged feeding (28 days) of foods can, at times, be poorly accepted by rats and result in lower food intake vs. the control and an underestimation of PER values. This is exemplified in the Canadian Food Inspection Agency’s (CFIA’s) list of PER values, where lentils have a PER of 0.3 [18]. This abnormally low PER is likely a function of reduced food intake over the 28-day feeding period. Shorter term protocols are clearly preferred, from both an animal care and a cost perspective, to the established PER methodology.

### 3.2. Challenge 2: PER Values Are Not Additive and Unestablished for New Foods

PER values are not additive. Thus, foods that contain multiple ingredients that contribute to the protein content of the final product are technically required to undergo PER testing via a rat bioassay to carry a protein quality claim. Of the 47 PER values listed in the CFIA’s *Industry Labelling Tool*, PERs for processed food products exist for bread, macaroni and cheese, beef stew, beef salami, bologna, sausage, wieners, cheddar cheese and chicken frankfurters [18]. Therefore, an animal bioassay is required for all other foods to which a PER is unestablished, even if each of the protein-containing ingredients in the food have a PER value.

Limited availability of PER values has been recognized by CFIA, as it is indicated in their *Industry Labelling Tool* that alternative metrics of protein quality, such as PDCAAS, can be used as support for a protein nutrient content claim when related to casein [18]. For example, the CFIA outlines that PER values can be derived from PDCAAS by using the following formula [18].

PER_PDCAAS_ = PDCAAS × 2.5 (1)

PER_PDCAAS_ represents a PER value generated from the product of PDCAAS multiplied by 2.5—the corrected PER value for casein. This formula has not been validated as an appropriate algorithm for extrapolating the PER for food. Although the PDCAAS has the physiological advantage of relying on amino acid requirements derived from humans rather than growing rats, because the PER_PDCAAS_ equation simply provides an alternative method for deriving a PER, the limitations and concerns over its validity within regulatory frameworks persist.

### 3.3. Challenge 3: The PER Is Combined with the RDI for Generating the Protein Rating of a Food

As demonstrated in Figure 1, the PER is multiplied by the level of protein in the RDI to determine the protein rating and verify if a food qualifies for a protein content claim. According to the CFIA’s *Industry Labelling Tool*, the RDI for most foods is considered to be one average serving of the food [23] and stems from the observation that many foods are only eaten at one occasion in a single day. However, in the case of foods such as milk, bread or butter, where several servings may be consumed daily, an RDI has been estimated by considering the food habits of Canadians. In addition to the RDI, Health Canada has established reference amounts for numerous foods that serve as the basis for other nutrient content and health claims. Rather than a daily intake, the reference amount is a regulated quantity of food that is consumed during a single eating occasion [24]. Thus, while the reference amount is a serving of food that is consumed during a single eating occasion, the RDI of a food is the amount of the food that is consumed on a given day. To highlight the differences between the reference amount and the RDI, it is useful to compare the reference amount and RDI for milk, which are 250 mL [24] and 852 mL [17], respectively. Given that the PER is based on the amount of protein in the RDI, the protein rating for milk would be inflated and makes an assumption regarding an individual’s dietary pattern for a specific food for that given day.

For some foods, the RDI is less than the reference amount. A good example is high-density breakfast cereal (≥43 g/250 mL), for which the RDI is 28 g (without milk) [17] or 30 g (with milk) [16] and the reference amount is 55 g [24]. Table 1 lists the calculated protein rating for two hypothetical, but reasonable high density breakfast cereals. One is a high density soy-based breakfast cereal, and the second is a high density chickpea-based breakfast cereal. For both, it is assumed that each cereal contains 5 g of protein per reference amount (55 g), from soy and chickpeas, respectively. As seen in Table 1, for both the soy-based and chickpea-based cereals, the highest achievable protein rating is 5.9 (using the RDI of 28 g), which is 71% below the required protein rating threshold of 20 to claim, at minimum, a “good source” of protein in Canada. As per the Canadian *Food and Drug Regulations*, the addition of 125 mL milk can be factored into the protein rating calculation, where the RDI for the breakfast cereal is adjusted to 30 g [16]. However, even with the addition of milk, the highest achievable protein rating would be 17.1 and still remains below the threshold for a protein content claim. Interestingly, if the 55 g reference amount for a high density breakfast cereal was permitted to be used in the protein rating calculation and it is assumed that that 125 mL remains as the amount of milk consumed with the breakfast cereal, both the soy-based and chickpea-based breakfast cereals would be considered a “good source” of protein.

The application of RDIs to quantify the protein rating of a food is an illogical approach to protein content claims. Similar to micronutrient content claims, protein content claims should refer to the characteristics of the food that is consumed at the specific eating occasion, not the amount of the food that is assumed to be consumed over the course of the day.

A more rational approach would be to have protein claims based on the protein contribution of a food relative to the reference amount, which, again, is a regulated quantity of food that is consumed during a single eating occasion. Not only would this complement the Nutrition Facts Table, but also allow Canadians to use protein content claims as a tool for making healthy dietary choices.

## 4. Proposed Regulatory Frameworks for Modernizing Regulations for Protein Content Claims in Canada

Communication efforts that promote food products that contain significant levels of plant-based proteins are largely disadvantaged because of the inability to meet Canada’s regulatory standards for protein quality. Scientists and policy makers are encouraging an increase in the incorporation of plant-based foods into dietary paradigms as a means to promote sustainable food systems, reduce the prevalence of diet-related diseases and manage costs linked to healthcare and loss of productivity. This is likely due to the growing amount of evidence that suggests a whole food plant-based diet that includes plant-based protein helps to manage the risk of diet related conditions such as obesity, cardiovascular disease, type 2 diabetes, high blood pressure and high cholesterol [26,27,28,29,30,31,32,33,34]. In addition, the 2015–2020 Dietary Guidelines for Americans also cite a healthy vegetarian eating pattern as one of three dietary patterns to help promote health and prevent chronic disease in the USA [35]. Consumers require access to this detail as health and weight loss are a driving interest in selecting meat alternatives, with 30% consuming these products because they are watching their cholesterol, 29% because of saturated fat, and 28% for weight loss [36]. Therefore, stakeholders of the food system should be given the appropriate tools and regulatory frameworks to align with human health and environmental targets. The following sections of this commentary attempt to provide solutions that would assist to provide and communicate the benefits of a wider variety of protein-containing foods to Canadians.

### 4.1. Option 1 (Preferred Option): Permit Protein Content Claims Made in Food Labeling and Advertising in Canada to Be Based on the Absolute Protein Content

Canada’s food system provides substantial dietary variety that permits the adoption of a spectrum of healthy dietary patterns that are omnivorous or characterized by various degrees of vegetarianism. Given that protein from plant-based foods is often considered to be of lower quality, an assessment of dietary quality among vegetarian populations in developed regions is a reasonable approach for determining whether higher consumption of plant-based foods results in protein and/or amino acid insufficiency. It is well known that adoption of a vegetarian-type diet, particularly a vegan diet, can provide some nutritional challenges that are largely derived from a decrease in the consumption of animal-derived foods such as such as vitamin B12, vitamin D, iron and zinc [37]. However, few studies, if any, provide evidence to support that, within well-developed food systems, vegetarian diets lack sufficient protein or indispensable amino acids.

In a cross-sectional study conducted in Belgium, omnivorous diets were compared to varying degrees of vegetarian diets [38]. Based on data from food-frequency questionnaires, it was demonstrated that a vegan diet was highest in dietary fiber and lowest in sodium. While protein consumption was lowest in vegan diets (82 g/day) compared to omnivorous diets (112 g/day), total daily intake of protein was adequate. Vegan diets were comprised of a variety of protein sources, including legumes and nuts, that, when combined with cereal can provide adequate levels of indispensable amino acids and non-essential nitrogen. Moreover, compared to all other dietary patterns examined, vegan diets scored highest on the Healthy Eating Index [38]. Although intakes of specific amino acids were not evaluated, amino acid insufficiency was also not identified as a concern [38]. Similarly, in a comparison of three-day diet records and nutritional status biomarkers between Finnish vegans and non-vegetarians, it was observed that protein intake was significantly lower among vegans (74 g/day vs. 103 g/day; 13.7% of energy vs. 19.1% of energy), but was not identified as a concern [39]. Compared to non-vegetarians, vegans had higher intakes of legumes, tofu and soy beverages [39]. Recently, as part of the European Prospective Investigation into Cancer and Nutrition (EPIC)-Oxford cohort, Schmidt et al. [40], compared plasma concentrations and dietary intakes of amino acids in male meat-eaters, fish-eaters, vegetarians, and vegans. While plasma concentrations were generally lowest in vegans, the differences were not as substantial as one would have expected based on dietary amino acid intakes [40]. The recent data from Clarys et al. [38], Elorinne et al. [39] and Schmidt et al. [40] demonstrated that nutritional challenges associated with plant-based diets are predictable in the developed world and protein or amino acid insufficiencies are not of concern within the general population.

The application of protein quality in determining the eligibility of a food for a protein content claim insinuates that, on any given day, the protein consumed at every eating occasion should provide a minimum level of indispensable amino acids, or in the case of the PER, facilitate a minimum growth rate among weanling rats. However, while the indispensable amino acid composition of pulses and cereals are complementary when consumed together, there is no evidence to support the notion that all indispensable amino acids need to be consumed at every meal. That is, protein and amino acid requirements can be met throughout differing meal intakes within a given day [41]. This is somewhat reflected in regulatory frameworks in Europe, Australia and New Zealand, China, and South Korea, where, rather than protein quality, absolute levels of protein per serving of a given food are used to support protein content claims (Table 2). In Europe, “source of”’ and “high source” of protein claims can be made on foods that provide at least 12% and 20% energy from protein per serving, respectively [10]. In Australia and New Zealand, where food standards established by Food Standards Australia New Zealand are used by both jurisdictions, a general protein claim can be made for foods that contain ≥5 g protein/serving, while a “good source” of protein claim can be made for foods that contain ≥10 g protein/serving [11]. The regulatory frameworks for China and South Korea are similar to the International Food Standards put forth by Codex Alimentarius where a food is considered a source of protein if it provides ≥10% of the Nutrient Reference Value (NRV) for protein per 100 g, or ≥5% of the NRV per 100 mL, or ≥5% of the NRV per 100 kcal (Codex [42] & South Korea [13]) or 420 kJ (China [12]). For Codex and South Korea, foods that are ≥10% of the NRV per serving are also considered to be a source of protein [13,42]. For all three regions, a “high source” of protein is reserved for foods that contain 2x the level of protein that qualifies for a “source” claim [12,13,42]. The protein NRV for Codex [43], South Korea [13], and China [12], is 50 g, 55 g, and 60 g, respectively.

Codex’s framework for protein content claims is an internationally recognized standard and demonstrates consensus for the use of total protein levels as support for protein content claims. Given Canada’s similarity to Europe, and Australia and New Zealand, where sophisticated food systems provide the population with a plethora of affordable foods of both animal- and plant-origin, it is not likely that adoption of a similar system in Canada would lead to widespread deficiency of indispensable amino acids. In developed regions, studies have yet to show that not using protein quantity to support protein content claims is negatively associated with the misrepresentation of foods, lower quality of foods, or diets within their respective marketplaces, and more broadly, the protein status of their respective populations. Similarly, to our knowledge, there are no data to suggest that the general population in Canada is at risk for total nitrogen or indispensable amino acid insufficiency. Dietary survey data in Canada show that protein intake is sufficient with < 3% of protein intakes among children, adolescents and adults being below the acceptable macronutrient distribution range [44,45,46]. To mitigate consumer confusion and permit straightforward comparisons between foods, 5 g and 10 g of protein per reference amount would be reasonable thresholds for “good source” and “excellent source” of protein claims in the labeling and advertising of foods in Canada (Table 3, Option 1); which aligns with Australia and New Zealand. Table 4 demonstrates the application of this suggestion, where the breakfast cereal from Table 1 containing 5 g of protein from chickpeas would not qualify for a protein content claim using the current regulatory system. However, if proposed Option 1 from Table 3 were adopted in Canada, the same food would be considered a “good source” of protein. With regulatory modernization and the constant evolution of Canada’s food landscape, removal of regulatory hurdles that seemingly impede consumer communication around the nutritional merits of food would help increase dietary inclusion of plant proteins, encourage positive dietary changes, and support goals regarding the sustainability of the Canadian agri-food industry.

### 4.2. Option 2 (Less Preferred): Adoption of PDCAAS as the Official Method of Protein Quality Assessment as Support for Protein Content Claims in Food Labeling and Advertising in Canada

Compared to PER, PDCAAS values can be theoretically calculated for foods with multiple ingredients that provide protein [9,22]. Also, although total fecal nitrogen digestibility values are typically determined using non-ruminant animal models, newer data also suggest that total fecal digestibility for foods can be estimated by in vitro methodologies [49,50,51], which could further expedite food innovations that contain non-animal protein sources.

Canada and the USA have similar food landscapes, therefore, Canada’s adoption of PDCAAS would serve as a reasonable interim solution to regulatory modernization for protein content claims. The following describes a suggested framework for using PDCAAS as the basis for protein content claims in Canada.

Box 1 **Suggested framework for adopting PDCAAS in Canada as support for protein content claims on food.**

1. Adopt PDCAAS as the official methodology for assessing the quality of the protein in foods sold in Canada.
2. Adopt 50 g as the DV for protein in Canada. This is the same threshold used in the USA for children ≥4 years of age through to adults [19].
3. Base protein content claims in Canada on the proportion of quality protein (corrected by PDCAAS) found in a food relative to the DV for protein (50 g).
4. Protein claims in Canada are currently based on RDIs. Rather than RDIs, it is suggested that protein content claims be based on reference amounts, which corresponds to RACCs in the USA. This permits protein quality and the corresponding claim to be directly applicable to how a food is consumed relative to the DV for protein. This also permits consumers to better quantify the contribution of protein in a food to daily protein requirements and aligns with the basis for other nutrient content claims, as well as how the DV is calculated for other Nutrition Facts Table.
5. Permit the utilization of in vitro methodologies to determine the total protein digestibility of foods with unestablished PDCAAS values. In vitro determination of total protein digestibility values will expedite innovation and reformulation of food products. To permit the use of such methods, it is reasonable that Health Canada identify one or more acceptable methods for generating digestibility values in vitro as this would help limit variability and permit comparisons across foods.
6. Adopt a three-tier claim framework for protein. The proposed thresholds are summarized in Table 3 (Option 2) relative to a 50 g DV for protein (see criteria 3). Under the current Canadian framework, “source of” and “good source of” protein claims are considered equivalent [16]. However, proposed Option 2 outlined in Table 3 aligns with requirements for nutrient content claims for dietary fibre, vitamins, and minerals where “source,” “good source,” and “excellent source” claims are of increasing magnitude [52]. Furthermore, a three-tier system creates more opportunities for the protein content in plant-based protein sources to be promoted as a nutritional attribute of the food. Under Option 2, minimum levels of corrected protein per reference amount for each tier of protein content claim would be 2.5 g, 5 g and 10 g protein, respectively. A threshold of ≥5 g protein/reference amount for a “good source” of claim and ≥10 g protein/reference amount for an “excellent source” claim aligns with the USA framework when a 50 g DV for protein is used to support protein claims for children ≥4 years of age through to adulthood [19]. A 2.5 g protein/reference amount limit was derived from the current Canadian framework where a 5% DV for vitamins, minerals, and fibre represents the threshold for “source of” claims [52].
7. When two or more distinct foods are traditionally consumed together, it is proposed that the sum of their corrected protein levels can be used to calculate the %DV as support for a protein content claim. This consideration is already in use in the Canadian *Food and Drug Regulations* where the protein rating for breakfast cereal can be combined with the protein rating for 125 mL milk [16]. Again, Table 4 provides an example where the chickpea-based breakfast cereal from Table 1 would qualify for a “good source” of protein content claim under the proposed PDCAAS system outlined herein. Conversely, this same cereal would not qualify for the same claim within the current regulatory system using the PER and protein rating system.
8. It is understood that protein requirements (mg/g protein) for a reference population used to calculate the PDCAAS of a food can change as data corresponding to specific age-sex groups continue to evolve. Therefore, the reference population and associated indispensable amino acid requirements (mg amino acid/g protein) should be incorporated by reference. This would permit Health Canada to make expedited changes to the regulatory framework for PDCAAS-based assessments of protein quality for foods.

In addition to the principles that directly complement the regulatory structure in the USA, criteria five, six, seven, and eight, misalign with the USA to better reflect Canada’s evolving food system and considers how foods are consumed within the Canadian marketplace.

While PDCAAS has been discussed in the context of a more accurate measure of protein quality that aligns with the USA, an updated version has emerged to address inaccuracies in the original PDCAAS calculation. Although the USA regulations cite the Joint FAO/WHO 1991 report on Protein Quality Evaluation methodology [9] for calculating PDCAAS for foods [19], the PDCAAS methodology was updated in the FAO/WHO Joint 2007 report on Protein and Amino Acid Requirements in Human Nutrition [22]. The updated report outlines that PDCAAS should be calculated by first determining the weighted average of digestible amino acid content for each ingredient and, second, applying the weighted average protein digestibility coefficient. By not applying the weighted average digestibility amino acid content, there is risk in generating inaccurate PDCAAS values. If Canada were to adopt PDCAAS, which version best accommodates the needs of the Canadian food system requires further assessment.

## 5. Discussion

Regulatory frameworks that help facilitate dietary change for the population are an important tool for enabling Canadians to make positive dietary choices. In this commentary, limitations of the PER and protein rating method were detailed. The methodological shortcomings of the PER and corresponding protein rating system support the request that Health Canada revise the current regulatory framework for protein content claims for foods. Ideally, for the purposes of labeling or advertising a food with a protein content claim, it is suggested that the requirement to demonstrate the quality of a protein for a content claim be replaced with a framework that requires the absolute level of protein in the food. These changes would serve to modernize the regulatory framework in Canada, expedite food innovation, and, most importantly, allow for communication initiatives that educate Canadians on the contribution of plant-based foods to meeting protein requirements. Alternatively, if Health Canada wishes to keep protein quality as a qualifying criterion for protein messaging, adoption of PDCAAS would likely be the most efficient interim solution. Albeit not perfect, the use of PDCAAS in Canada would address some of the challenges that the PER method presents within the Canadian food environment, including harmonization with the USA. Given that total digestibility could be determined in vitro under the proposed framework discussed in Section 4.2 and the PDCAAS for mixed foods can be theoretically generated, the PDCAAS-based framework discussed in this commentary would reduce some of the barriers that slow food innovation and reformulation in Canada.

The removal of protein quality as support for protein content claims has been positioned as the preferred option for updating Canadian regulations. As previously discussed in Section 4.1, there are other developed regions that do not use protein quality as support for communicating the presence of protein in foods. Although data have yet to demonstrate adverse outcomes in Europe, Australia and New Zealand, China, and South Korea secondary to the protein quality of the food landscape, as with any regulatory or policy change, due diligence on the part of the Canadian government would be required to ensure that the safety of Canadians would not be compromised if protein content claims were based on absolute levels of protein. Similarly, if protein quality were to be removed from protein content claims, additional requirements or conditions may be required to prevent the addition of food ingredients that contain adequate protein levels, but high levels of single dispensable amino acids or significant imbalanced amino acids to foods as a means to enhance the protein content with little effect on dietary quality.

Of the two proposed options for protein contents claims, there is a rationale for maintaining a two tier claim system if protein quality is to be removed from regulations vs. the adoption of a three tier claim system if the proposed PDCAAS framework was adopted in Canada (Table 3). For the former, having higher thresholds for protein content per reference amount at least partially accounts for possible disparities in levels of indispensable amino acids. Conversely, because PDCAAS accounts for levels of indispensable amino acids, it is reasonable that cut-offs for protein content claims start at a lower limit, i.e., ≥2.5 g protein (or ≥5% DV)/reference amount.

As it currently stands, Canada’s regulatory framework for protein content claims remains a barrier for food innovation and/or reformulation to include plant-based and sustainable sources of protein. One of the fundamental hindrances of the PER method is the lack of PER values. Unless manufactures are willing to invest in determining the PER of a new food, healthful foods may not be launched into the Canadian marketplace as they will be unable to communicate that they can provide a reasonable amount of “quality” protein per serving to the diet. Table 5 provides a summary of four existing foods in the Canadian marketplace that have been recently reformulated to include increased levels of plant-based protein. With the exception of the pancake mix and bread (RDI of 5 slices/day), the breakfast cereal and pasta did not qualify for a protein content claim under Canada’s current regulatory framework. However, under both proposed frameworks outlined in this review, all of the food products would quality for either a “source,” “good source,” or “excellent source” of protein claim. If anything, the use of a protein quality rating system that helps to facilitate innovation and nutrition literacy can enhance Canadian’s ability to integrate nutrient dense foods into omnivorous and vegetative diets. It is also relevant to emphasize that, outside of Canada and the USA, protein content claims are not contingent on protein quality. One could argue that regulations in Australia and New Zealand, Europe, China, and South Korea serve as a “proof of concept,” which altogether questions the need for protein quality as support for corresponding nutrient claims. As dietary guidelines across jurisdictions emphasize variety, protein from an array of foods can be consumed throughout the day to meet requirements for indispensable amino acids. Again, the notion that each eating occasion at which protein is consumed should contain a specific threshold of quality is, to our knowledge, unsupported by the literature [41]. This rationale is analogous to governance of therapeutic health claims in Canada where required daily dosages of a nutrient or ingredient that correspond to a physiological benefit can be consumed throughout a given day. For example, a cholesterol-lowering health claim for soy protein was approved by Health Canada in 2015. Although 25 g/day soy protein supports reductions in low-density lipoprotein (LDL)-cholesterol levels, to make a claim, foods are required to contain a minimum of 6 g soy protein/serving.

The CFIA has acknowledged the shortcomings of the PER/Protein rating system by providing an equation that generates PER values from the PDCAAS of a given food [18]. While this is viewed as a positive development within the tools provided by the CFIA, it is not a long-term solution as the approach has not been validated and perpetuates uncertainty and risk among industry stakeholders for communicating the presence of protein in food products that do not have an established PER value.

The purpose of any nutrient content claim is to guide the consumer to foods that can contribute macro- or micronutrients to the diet. Nutrient content claims should also facilitate dietary choices that best align with dietary guidance that promote variety as well as address global challenges around food production and dietary patterns. Of the two proposed approaches in this commentary, removal of protein quality (Table 3, Option 1) as a criterion for supporting protein content claims would be a progressive option to help to accelerate progress in response to the challenges linked to diet and food production in Canada. Ongoing study and use of protein quality as a tool remains valid as optimized consumption of complementary plant-based protein sources on any given day remains important for consumers. Also, while the need for protein content claims to be based on protein quality is questioned within this commentary, prescriptive use of metrics of protein quality in the context of meals likely have has better utility in specific applications such as medicine or athletic performance. Furthermore, although the theme of sustainability is beyond the scope of this commentary, it is worthwhile to acknowledge the ongoing global strategies that consider the impact of dietary choices on metrics of sustainability within food systems [7]. Regulatory frameworks affect consumer’s ability to choose foods that assist in shifting to dietary patterns that consider nutrition, health and sustainability. Dietary guidelines in other jurisdictions, such as Brazil and the Nordic Countries, are already linking nutritional adequacy with the environmental impact of dietary choices, specifically as they pertain to dietary protein across a spectrum of vegetarian and omnivorous dietary paradigms [3,53]. Recently, Ruin et al. [54] demonstrated that omnivorous diets that increase reliance on plant-based foods, can have a profound effect on the carbon, water and ecological footprint of the diet. A regulatory framework that promotes innovation, communication and return-on-investment should align with initiatives that support a food system that encompasses both health and sustainability.

## 6. Conclusions

This commentary provided a synopsis of the challenges of Canada’s reliance on the PER and protein rating system for aligning with efforts to increase the use plant-based sources protein in the diet. Two proposed approaches to regulatory modernization for protein content claims were provided. The first and preferred approach (Option 1) calls for the removal of protein quality for identifying foods as a “source” of protein. Utilization of absolute thresholds of protein (g) could harmonize with Australia and New Zealand, where 5 g and 10 g protein per reference amount as cut-offs for “good source” and “excellent source” protein claims, respectively, would be a reasonable avenue for Canada. Positioned as a less desirable, but acceptable interim solution, the second proposal (Option 2) was for Canada’s adoption of a PDCAAS-based framework and a protein DV of 50 g. A suggested framework for implementing PDCAAS for supporting protein content claims included expanding protein content claims from two to three levels of magnitude (“source,” “good source,” and “excellent source”) (Table 3, Option 2) and allowing total protein digestibility values to be generated using in vitro methods. These two conditions permit the agility required to accelerate the development of food platforms that support the increased use and promotion of plant-based protein sources. More broadly, both proposed regulatory structures outlined in this commentary assist in removing the barriers that prevent stakeholders from addressing the health and environmental challenges that are linked to lifestyle and food production, respectively. While food regulations are intended to protect the public from exposure to misleading information on food labels, they are currently acting as an obstacle to meeting benchmarks linked to healthy and sustainable diets. Together, modernization of Canada’s regulatory framework around protein content claims is a formidable step to providing Canadians with the tools required to make inclusive decisions about food choices and realize the capacity to which a wide variety of foods contribute protein to the diet. After all, this aligns with the mandate of Canada’s Food Guide and other healthy dietary patterns.

## Figures and Tables

**Table 1:** Examples of the theoretical application of the PER and the use of the RDI vs. RA to determine the protein rating of specific foods in Canada.

Food	Description	RDI or RA	Protein (g) per RDI or RA	PER [18]	Protein Rating
Soy-based Breakfast cereal	◾ High density breakfast cereal: ≥43 g/250 mL ◾ 5 g protein from soy per 55 g reference amount ^‡^	◾ RDI: 28 g (alone) * ◾ RDI: 30 g (with milk) ^†^ ◾ RA: 55 g (alone) ^‡^	2.5	2.0	5.0
			2.7	2.0	5.4
			5.0	2.0	10.0
Chickpea-based Breakfast cereal	◾ High density breakfast cereal: ≥43 g/250 mL ◾ 5 g protein from soy per 55 g reference amount ^‡^	◾ RDI: 28 g (alone) * ◾ RDI: 30 g (with milk) ^†^ ◾ RA: 55 g (alone) ^‡^	2.5	2.32	5.8
			2.7	2.32	6.3
			5.0	2.32	11.6
Milk	◾ 2% MF	◾ RDI: 125 mL (with breakfast cereal) ^†^	4.3 ^¥^	2.5	10.8
Soy-based breakfast cereal + Milk	◾ High density breakfast cereal: ≥43 g/250 mL ◾ 5 g protein from soy per 55 g reference amount ^‡^ ◾ 2% MF	◾ RDI: 30 g (with 125 mL milk) ^†^ ◾ RA: 55 g (with 125 mL milk)	-	-	16.2 (5.4 + 10.8)
			-	-	20.8 (10.0 + 10.8)
Chickpea-based breakfast cereal + Milk	◾ High density breakfast cereal: ≥43 g/250 mL ◾ 5 g protein from chickpeas per 55 g reference amount ^‡^ ◾ 2% MF	◾ RDI: 30 g (with 125 mL milk) ^†^ ◾ RA: 55 g (with 125 mL milk)	-	-	17.1 (6.3 + 10.8)
			-	-	22.4 (11.6 + 10.8)

Abbreviations: MF, milk fat; PER, protein efficiency rating; RA, reference amount; RDI, Reasonable Daily Intake. * Canada: RDI for breakfast cereal without milk = 28 g [17]. ^†^ Canada: RDI for breakfast cereal with 125 mL milk = 30 g [16]. ^‡^ Canada: RA for a high density breakfast cereal (≥43 g/250 mL) = 55 g [24]. ^¥^ Canadian Nutrient File [25].

**Table 2:** Summary of alternative regulatory frameworks and international foods standards (Codex Alimentarius) for protein content claims.

Australia and New Zealand [11]	Europe [10]	Codex Alimentarius [42], China [12], and South Korea [13]
General Protein Claim ≥5 g protein/serving.	“Source” of Protein ≥12% of energy/serving.	“Source” of Protein * ≥10% the NRV * per 100 g (solids); or ≥5% the NRV * per 100 mL (liquids); or ≥5% the NRV * per 100 kcal (Codex and South Korea) or 420 kJ (China); or ≥10% the NRV * per serving (Codex and South Korea)
“Good Source” of Protein ≥10 g protein/serving.	“High Source” of Protein ≥20% energy/serving.	“High Source” of Protein At least 2x the level of protein that qualifies for a “source” claim.

Abbreviations: NRV, Nutrient Reference Value. * NRV for Protein: Codex Alimentarius: 50 g/day [43]; China: 60 g/day [12]; South Korea: 55 g/day [13].

**Table 3:** Thresholds for protein content claims for proposed approaches to regulatory changes for protein content claims in Canada.

Proposed Option 1: Removal of Protein Quality (Preferred)		Proposed Option 2: Adoption of PDCAAS (Less Preferred)			
Protein Content (g) per Reference Amount	Protein Content Claim	Corrected Protein Requirement per Reference Amount per DV *			Protein Content Claim
5 g	“Good source” of protein	≥5% DV	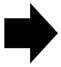	2.5 g	“Source” of protein
10 g	“Excellent source” of protein	≥10% DV		5 g	“Good source” of protein
		≥20% DV		10 g	“Excellent source” of protein

Abbreviations: DV, daily value; PDCAAS, protein digestibility corrected amino acid score. * Corrected for protein quality using PDCAAS. Proposed DV for protein in Canada is 50 g/day.

**Table 4:** An example that demonstrates the current and proposed approaches for qualifying for protein content claims in Canada when ≥2 distinct finished foods are traditionally combined prior to consumption.

Example from Table 1: Chickpea-based Breakfast Cereal and Milk (2% MF)		
◾ High density breakfast cereal (≥43 g/250 mL) = 55 g RA *		
◾ 5 g protein from chickpeas per 55 g RA		
◾ 125 mL milk (2% MF) = 4.3 g protein		
**Current Approach Using the PER ^†^**	**Proposed Option 1: Removal of Protein Quality** ^ɣ^ **(Preferred)**	**Proposed Option 2: Adoption of PDCAAS in Canada** ^ɣ^ **(Less Preferred)**
The food has a protein rating of 20 or more, as determined by official method FO-1, Determination of Protein Rating, October 15, 1981 ^‡^ (a) Per RDI; or (b) Per 30 g combined with 125 mL of milk, if the food is a breakfast cereal. ◾ PER_Chickpea_ = 2.32, PER_Milk_ = 2.0 [18] **1. Protein Rating _Breakfast Cereal_** $\;Protein\;rating_{Breakfast\;Cereal}=\;PER_{Chickpea}\;\times \frac{protein\;\left(g\right)}{RDI_{Breakfast\;Cereal}}\;$ $\;=2.32\;\times \frac{2.7\;g\;Protein}{30\;g\;Breakfast\;Cereal}=6.3\;per\;30\;g\;Breakfast\;Cereal$ **2. Protein Rating _Milk_** $\;Protein\;rating_{Milk}=\;PER_{Milk}\;\times \frac{Protein\;\left(g\right)}{RDI_{Milk}}\;$ $=2.5\;x\;\frac{4.3\;g\;Protein}{125\;ml\;Milk}=10.8\;per\;125\;ml\;Milk$ **3. Total Protein Rating** $\;Protein\;rating_{Breakfast\;Cereal+Milk}=6.3\;+10.8\;=17.1$ **4. Protein Claim** **Does not qualify for a protein claim**	◾ 5 g protein per RA = “Good Source” of protein. ◾ 10 g protein per RA = “Excellent Source” of protein. **1. Sum of protein in food per RA** 5 g proteinfrom chickpea+4.3 g protein from 125 ml milk=9.3 g protein per RA **2. Protein Claim** **Qualifies for a “good source” of protein claim**	◾ Protein per reference amount is corrected using the PDCAAS method ^§^ ◾ PDCAAS_Chickpea_ = 0.52 [47]; PDCAAS_Milk_ = 1.0 [48]. ◾ Proposed DV for protein in Canada: 50 g/day ◾ Assumed that 125 mL is the RA for milk when it is consumed with cereal. ◾ Protein claim is based on the level of corrected protein per RA relative to a protein daily value of 50 g: ≥5% DV (or 2.5 g/reference amount): “Source” of protein ≥10% DV (or 5 g/reference amount): “Good Source” of protein ≥20% DV (or 10 g/reference amount): “Excellent Source” of protein **1. Corrected Protein Level_Cereal_ per RA** $\;Corrected\;Protein\;Level_{Breakfast\;Cereal}=PDCAAS_{Chickpea}\times \frac{Protein\;\left(g\right)}{RA\;\left(g\right)}$ $\;=0.52\;\times \frac{5\;g}{55\;g\;RA}=2.6\;g\;protien\;per\;RA$ **2. Corrected Protein Level_Milk_ per RA** $\;Corrected\;Protein\;Level_{Milk}=PDCAAS_{Milk}\times \frac{Protein\;\left(g\right)}{RA\;\left(g\right)}$ $\;=1.0\;\times \frac{4.3\;g}{125\;ml\;RA}=4.3\;g\;protein\;per\;RA$ **3. Total Corrected Protein Level_Cereal + Milk_** $\;Total\;Corrected\;Protein\;Level_{Breakfast\;Cereal+Milk}=2.6+4.3$ =6.9 g **4. Protein Level Relative to the Proposed DV for Protein** $\;\%\;DV\;Protein=\;\frac{Protein\;level\;{\left(g\right)}_{Corrected}}{DV\;protein}$ $\;=\frac{6.9\;g}{50\;g\;protein}\times 100=\;13.8\text{\%}$ **5. Protein Claim** **Qualifies for a “good” source of protein claim**

Abbreviations: DV, daily value; MF; milk fat; PDCAAS, protein digestibility corrected amino acid score; PER, protein efficiency ratio; RA, reference amount; RDI, reasonable daily intake. * Government of Canada [24]. ^¶^ Canadian Nutrient File [25]. ^†^ Regulatory framework for protein content claims in Canada [16]. ^‡^ Government of Canada, Method FO-1 [14]. ^ɣ^ Proposed regulatory framework for protein content claims outlined in Table 3. ^§^ Food and Agriculture Organization of the United Nations [9].

**Table 5:** Summary and application of current and proposed regulatory frameworks for protein content claims for four existing foods recently reformulated to include increased levels of plant-based protein.

Food Innovation	Protein Ingredients	Current Canadian Framework *				Current USA Framework ^§^				Proposed Framework for Canada				
		Protein (g) per RDI ^†^	PER ^ɣ^	Protein Rating	Protein Claim (Y/N)	Protein (g) per RACC ^‡^	PDCAAS	%DV	Protein Claim (Y/N)	Proposed Option 1: Removal of Protein Quality ^Ÿ^ (preferred)		Proposed Option 2: Adoption of PDCAAS ^ǂ^ (Less Preferred)		
										Protein (g) per RA ^¥^	Protein Claim (Y/N)	PDCAAS	%DV	Protein Claim (Y/N)
1. Bread	Barley, Dry Navy Beans, cooked chickpeas, cooked lentils, yellow split peas, pinto beans, wheat gluten, soy protein, sunflower seed, whole wheat flour	17.7	1.15	20.3	Yes “Good Source”	7.1	0.458	6.5	No	10.6	Yes “Excellent Source”	0.458	9.7	Yes “Source”
2. Breakfast Cereal (Low Density: 20 g to 42 g/250 mL) ^¥^	Whole grain barley, whole grain wheat, pea protein concentrate	5.3	1.68	9.3	No	7.4	0.671	9.9	No	5.5	Yes “Good Source”	0.671	7.4	Yes “Source”
125 mL Milk	(2% MF)	4.3	2.5	6.8	N/A	4.3	1.00	8.6	N/A	4.3	N/A	1.00	8.6	N/A
Breakfast Cereal + 125 mL Milk^†^	Whole grain barley, whole grain wheat, pea protein concentrate, milk (2% MF)	N/A	N/A	16.1	No	N/A	N/A	N/A	N/A	9.8	Yes “Good Source”	N/A	16.0	Yes “Good Source”
3. Pancake Mix	Whole wheat flour, whey protein concentrate, pea protein, rice protein, soy flour, whole egg powder	20.7	2.25	46.7	Yes “Excellent Source”	30.4	0.901	54.8	Yes “Excellent Source”	20.8	Yes “Excellent Source”	0.901	37.4	Yes “Excellent Source”
4. Tricolour Pasta	Semolina, pea protein	12.6	1.37	17.3	No	8.1	0.549	8.9%	No	8.1	Yes “Good Source”	0.549	8.9%	Yes “Source of”

Abbreviations: %DV, percent daily value; N/A, not applicable; MF, milk fat; N, no; PDCAAS, protein digestibility corrected amino acid ratio; PER, protein efficiency ratio; RA, reference amount; RACC, reference amount customarily consumed; RDI, Reasonable Daily Intake; Y, yes. * Current Canadian Framework: A “good source” of protein claim is permitted when the protein rating is ≥20 per RDI; an “excellent source” of protein claim is permitted when the protein rating is ≥40 per RDI [16,55]; Protein Rating = PER x protein (g) per RDI [18]. ^†^ Canada, RDI: Bread, 5 slices (125 g) [17]; Breakfast Cereal, 30 g (with milk) [16]; Pasta, 85 g (dry) [17]; Milk (with breakfast cereal), 125 mL [16]; the RA is used to calculate the protein rating for pancakes because an RDI is not available ^¥^ [18]. ^ɣ^ With the exception of milk (2%), the PER for foods listed in Table 5 were determined using the following formula as outlined by the Canadian Food Inspection Agency: PER = PDCAAS × 2.5 [18]; the PER for 2% milk = 2.5 [18]. ^§^ Current USA Framework: Corrected protein level (using PDCAAS) per RACC; DV protein = 50 g/day [19]; a “good source” of protein claim is permitted when the corrected level of protein is ≥10% (5 g) the DV per RACC; an “excellent source” of protein claim is permitted when the corrected level of protein is ≥20% (10 g) the DV per RACC (10 g) [19]; %DV = [(PDCAAS × protein (g) per RACC)/50 g] × 100. ^‡^ USA, RACC: Bread, 50 g; low density Breakfast Cereal (20 to < 43 g per 240 mL), 40 g; Pancakes, 110 g; Pasta, 55 g (dry) [19]. ^Ÿ^ Proposed Option 1 (Table 3): A “good source” of protein claim is permitted when a food contains 5 g protein per RA; an “excellent source” of protein claim is permitted when a food contains 10 g protein per RA. ^¥^ Canada, RA: Bread, 75 g [24]; low density Breakfast Cereal (20 g to 42 g per 250 mL), 30 g [24]; Pancakes, 75 g [24]; Pasta, 55 g (dry) [24]. ^ǂ^ Proposed Option 2 (Table 3): Corrected protein level (using PDCAAS) per RA and adoption of 50 g/day as the DV for protein; a “source” claim is permitted when the corrected level of protein is ≥5% (2.5 g) the DV per RA; a “good source” claim is permitted when the corrected level of protein is ≥10% (5 g) the DV per RA; an “excellent source” claim is permitted when the corrected level of protein is ≥20% (10 g) the DV per RA. %DV = [(PDCAAS × protein (g) per RA)/50 g] × 100.
